# No difference in mid-term outcome after superior vs. anteroinferior plate position for displaced midshaft clavicle fractures

**DOI:** 10.1038/s41598-021-01625-4

**Published:** 2021-11-11

**Authors:** Philip-Christian Nolte, Anna-Katharina Tross, Julia Studniorz, Paul-Alfred Grützner, Thorsten Guehring, Marc Schnetzke

**Affiliations:** 1 Clinic for Trauma and Orthopaedic Surgery, BG Trauma Center Ludwigshafen at the University of Heidelberg, Ludwig-Guttmann-Strasse 13, 67071 Ludwigshafen, Germany; 2 Clinic for Orthopaedic Surgery, Heidelberg University Hospital, Schlierbacher Landstraße 200a, 69118 Heidelberg, Germany; 3 Department of Shoulder and Elbow Surgery, Sportsmedicine & Traumatology, Diakonie Clinic Paulinenhilfe, Rosenbergstraße 38, 70176 Stuttgart, Germany; 4 German Joint Centre, ATOS Clinic Heidelberg, Bismarckstraße 9, 69115 Heidelberg, Germany

**Keywords:** Medical research, Outcomes research

## Abstract

To compare outcomes, complications, revisions, and rates of implant removal of superior compared to anteroinferior plating in displaced midshaft clavicle fractures at mid-term follow-up. We retrospectively reviewed 79 patients who underwent operative treatment for displaced midshaft clavicle fractures (Group A: 28 patients with superior plating; Group B: 51 patients with anteroinferior plating) that were at least 2 years postoperatively. Adjusted Constant Score (aCS), Visual Analog Scale (VAS), and Quick Disabilities of the Arm, Shoulder and Hand (QuickDASH) score were compared. Bone union, implant removal, complications and revision surgeries were assessed. Group A had a significantly higher aCS compared to group B (90, IQR: 85.0–91.0 vs. 91, IQR: 90.0–93.0; *P* = 0.037). No significant differences between groups were seen in VAS (*P* = 0.283) and QuickDASH (*P* = 0.384). Bone union was achieved in 76 patients (96.2%) with no significant differences between groups (Group A: 96.4% vs. Group B: 96.1%; *P* > 0.999). There were no significant differences in implant removal rates (Group A: 60.7% vs. Group B: 66.7%; *P* = 0.630), complications (Group A: 46.4% vs. Group B: 31.4%; *P* = 0.226) and revisions (Group A: 25% vs. Group B: 9.8%; *P* = 0.102). Superior and anteroinferior plating result in high bone union rates and good clinical outcomes with similar rates of plate removal.

## Introduction

Open reduction and internal plate fixation has provided excellent functional and radiographic outcomes after midshaft fractures of the clavicle^1–3^; however, plate positioning remains a subject of controversy^4–7^. Plates can be implanted in either the classic superior position or in the anteroinferior position.

The anteroinferior plate position offers some potential benefits including less prominence and better soft tissue coverage compared to superior plates^4,8,9^. Patients with a plate on the superior aspect of the clavicle often complain about localized symptoms of pain and discomfort and eventually undergo hardware removal^10^. In contrast, reduced implant removal has been demonstrated following anteroinferior plating^8,11^.

It is known that the subclavian vessels^8,12^, brachial plexus^13^ and lungs^14^ are at risk during surgery due to their proximity to the clavicle. Advocates of anteroinferior plating claim that the trajectory of the drill bit is aimed posterosuperiorly; thus, aimed away from neurovascular structures^9,15^.

Superior plating of midshaft clavicle fractures is a procedure that has been performed frequently in the past with good functional results and low non-union rates^2,3,16^. Compared to anteroinferior plating, superior plating has been described as the easier technique resulting in shorter operation times^8,17^. Biomechanical studies have demonstrated high stability of superior plating constructs with superior stiffness in axial compression, torsion, and bending loads to failure compared to anteroinferior plates^18–20^. Although many authors see a potential benefit in using anteroinferior plates for the aforementioned reasons, thus far, the literature does not delineate clear indications^4,7,8^. Furthermore, the currently available literature is relatively limited and primarily looks at only short-term follow-up of less than 4 years^8,11,21,22^. Longer-term follow-up is especially relevant for determining the rates of implant removal. It is usually recommended to leave the plate for at least 1 year following fracture fixation; thus, studies with short-term follow-up may underestimate implant removal rates.

Therefore, the purposes of this study were to compare outcomes, complications, revisions, and rates of implant removal of superior compared to anteroinferior plating in displaced midshaft clavicle fractures at mid-term follow-up. It was hypothesized that anteroinferior plating of displaced midshaft clavicle fractures would result in superior clinical outcomes, fewer complications and lower implant removal rate when compared to superior plating.

## Materials and methods

### Study design

**Figure 1:**
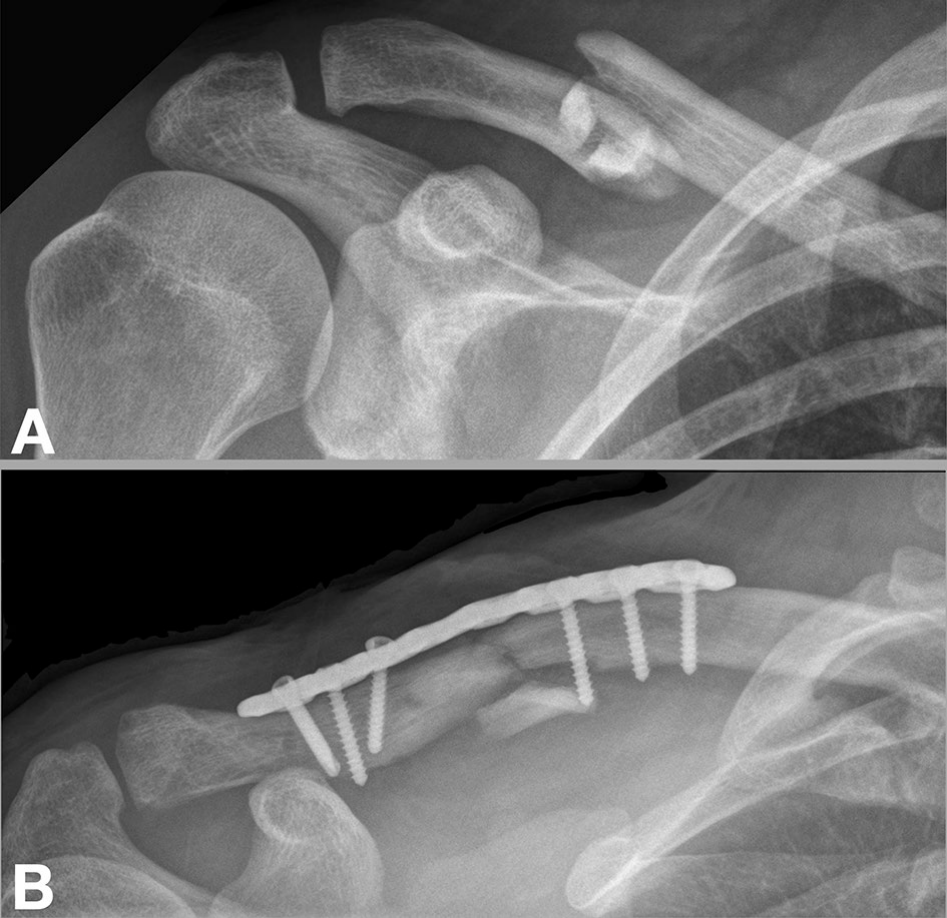
Radiographic anteroposterior images (right shoulder) of a 30-year-old female following a motorcycle accident demonstrating a midshaft clavicle fracture and fracture fixation with a superior plate. (**A**) Displaced midshaft clavicle fracture, (**B**) plate fixation with a superior plate.

**Figure 2:**
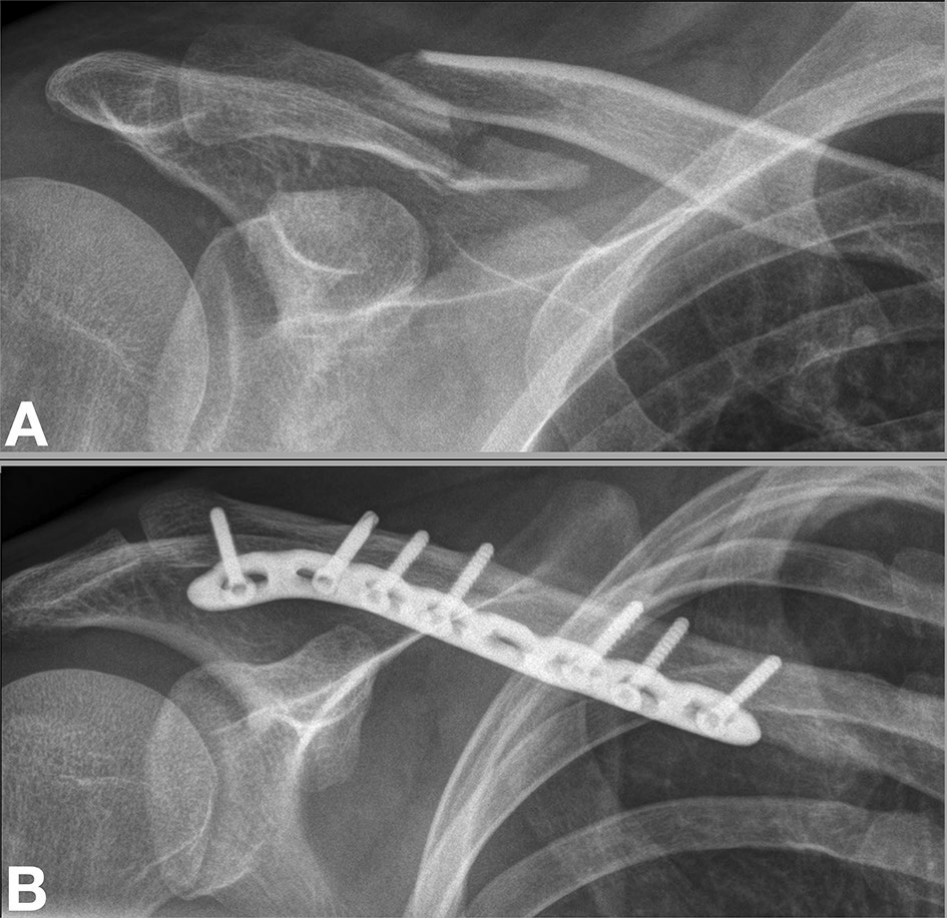
Radiographic anteroposterior images (right shoulder) of an 18-year-old male following a fall while playing soccer demonstrating a midshaft clavicle fracture and fracture fixation with an anteroinferior plate. (**A**) Displaced midshaft clavicle fracture, (**B**) plate fixation with an anteroinferior plate.

Ethics committee approval was obtained prior to data collection and informed consent was obtained from all patients included. In this retrospective cohort study, all patients with acute, displaced midshaft clavicle fractures were operatively treated at a level-1-trauma center between May 2009 and November 2014 were included if they met the following inclusion criteria: displaced clavicle fractures type 2B according to Robinson^23^ (Figs. 1, 2), treatment within 4 weeks after trauma^22^, patient age at time of surgery ≥ 18 and ≤ 80 years, operative treatment with either superior or anteroinferior locking compression plates (LCP), and were at least 2-year postoperatively. Patients were excluded if they had an open fracture, a prior ipsilateral clavicular fracture, and if they were not able to adhere with the postoperative protocol. This led to a total of 79 included patients. Patients treated with superior plating (group A) were compared to those with anteroinferior plating (group B). All surgeries were performed by one out of four board certified orthopedic trauma surgeons with extensive experience with this procedure. Until early April 2010, the standard plate configuration at our institution was superior plating. In late April 2010 the standard plate configuration was changed to anteroinferior plating as this was believed to result in less implant removal rates without compromising healing and stability.

### Operative procedure

#### Superior plate fixation

Following the induction of general anesthesia, the patient was placed in the beach-chair position and was prepped and draped in sterile orthopedic fashion. A 10 cm incision was made directly over the superior aspect of the clavicle. Subcutaneous dissection was carried out. Small vessels were cauterized throughout the procedure. The superior aspect of the clavicle was then exposed. Fracture fragment ends were identified, repositioned and held in place with fracture clamps or K-wires. If possible, a lag-by-application technique was used perpendicular to the fracture line for interfragmentary compression. When the fracture was reduced anatomically, an appropriately sized locking compression plate (LCP, DePuy Synthes, MA, USA) was contoured to the superior aspect of the clavicle and filled with locking screws. Additionally, cortical screws were placed on the outer sides of the plate and on each side of the fracture, respectively, according to the AO technique (Fig. 1). Two fluoroscopic views were used to demonstrate reduction of the clavicle and position of the implant. Finally, the wound was thoroughly irrigated with saline before fascia and skin were closed in layers.

#### Anteroinferior plate fixation

The approach to anteroinferior plating only differed from that of superior plating in that the incision was made slightly anterior to the clavicle and the locking compression plate (LCP, DePuy Synthes, MA, USA) was positioned onto the anteroinferior aspect of the clavicle (Fig. 2). This plate position allowed for the placement of long screws from anteroinferior to posterosuperior, especially in the lateral aspect of the clavicle.

### Postoperative rehabilitation

The operative extremity was placed in a sling for 2 weeks and early active range of motion of the elbow, hand, and wrist was encouraged immediately postoperatively. Passive range of motion of the shoulder joint to 90° abduction/flexion was started on the first postoperative day. Active-assisted and active range of motion was begun after the wound had healed and was restricted to 90° abduction/flexion for 6 weeks. Both groups received the same standard postoperative rehabilitation.

### Outcome parameters

Patient medical records were reviewed for baseline characteristics. Demographic data was obtained including age, sex, hand dominance, dominant side injured, time to surgery, and polytrauma. Injury mechanism and plate-related complications and further dependent surgical interventions were recorded.

At a minimum 2-year follow-up, patient-reported outcome scores were collected including the age and gender adjusted Constant score (aCS)^24^, Quick Disabilities of the Arm, Shoulder and Hand (QuickDASH) score, and Visual Analog Scale (VAS) at rest and under stress. The aCS was chosen as the primary outcome measure. Bony healing was assessed on the most recently obtained radiographs and was defined as an invisible fracture line or bridging callus across the fracture line. In addition to the aforementioned scores, patients completed questionnaires regarding implant removal, time to implant removal, and return to work/previous workplace.

### Statistical analysis

Statistical analysis was performed with PRISM version 8.4.0 (GraphPad, San Diego, CA, USA). Categorical data are presented as number and percentages. For continuous data, mean and standard deviation (SD) were used if the data was normally distributed, and median and interquartile range (IQR) if the data was non-normally distributed. The independent t test was used for univariate analysis for normally distributed data and the Mann–Whitney test for nonparametric data. Bivariate data was analyzed with the Fisher exact test. Subgroup analyses were performed for implant removal rates of male and female patients. The level of significance was set at P < 0.05.

### Ethics approval

Ethics committee approval was obtained prior to data collection [Local Ethics Committee of Rhineland-Palatinate, Germany #837.485.16 (10804), July 22nd, 2016]. This study has therefore been performed in accordance with the ethical standards laid down in the 1964 Declaration of Helsinki and its later amendments.

### Consent to participate

All patients gave written informed consent prior to inclusion in the study.

### Consent for publication

Data collection, coding, routing and analysis were in accordance with legal data protection policy. Participants gave written consent for analysis and publication.

## Results

A total of 79 patients were included in this study. The study population was comprised of 63 men (79.7%) and 16 women (20.3%) with a mean age of 48.5 ± 13.0 years.

Patient demographics for each study group are summarized in Table 1. The groups did not differ significantly in age, injured side, hand dominance, dominant-sided injury, polytrauma, and time to surgery; however, there was a significant difference between groups regarding gender (group A: 18 (64.3%) vs. group B: 45 (88.2%); *P* = 0.018).

**Table 1:** Demographics of the patient cohort.

Demographics	Total	Superior	Anteroinferior	*P* value
Age, mean ± SD, y	48.5 ± 13.0	47.4 ± 13.8	49.2 ± 12.6	0.567*
**Gender, n (%)**				
Male	63 (79.7)	18 (64.3)	45 (88.2)	**0.018**^**†**^
Female	16 (20.3)	10 (35.7)	6 (11.8)	
**Injured side, n (%)**				
Right	32 (40.5)	9 (32.1)	23 (45.1)	0.339^†^
Left	47 (59.5)	19 (67.9)	28 (54.9)	
**Dominant side, n (%)**				
Right	70 (88.6)	27 (96.4)	43 (84.3)	0.147^†^
Left	9 (11.4)	1 (3.6)	8 (15.7)	
**Dominant side injured, n (%)**				
Yes	39 (49.4)	11 (39.3)	28 (54.9)	0.241^†^
No	40 (50.6)	17 (60.7)	23 (45.1)	
**Polytrauma, n (%)**				
Yes	22 (27.8)	10 (35.7)	12 (23.5)	0.298^†^
No	57 (72.2)	18 (64.3)	39 (76.5)	
Time to surgery, median (IQR), d	6 (4–9)	6.0 (3.0–8.8)	6.0 (4.0–10.0)	0.479^#^

Significant values are written in bold text.

IQR: Interquartile range, SD: standard deviation, *: Independent t test, ^†^: Fisher exact test, ^#^: Mann–Whitney test.

Injury mechanisms are summarized in Table 2. The most common mechanisms of injury were bicycle accidents (40.5%), followed by motor vehicle accidents (25.3%).

**Table 2:** Injury mechanisms.

Injury mechanism	Total	Superior	Anteroinferior
Bicycle accident, n (%)	32 (40.5)	10 (35.7)	22 (43.1)
Motor vehicle accident, n (%)	20 (25.3)	9 (32.1)	11 (21.6)
Sport accident, n (%)	9 (11.4)	3 (10.7)	6 (11.8)
Falls from ≤ 1.5 m, n (%)	9 (11.4)	2 (7.1)	7 (13.7)
Falls from > 1.5 m, n (%)	7 (8.9)	3 (10.7)	4 (7.8)
Other, n (%)	2 (2.5)	1 (3.6)	1 (2.0)

Time to follow-up and outcome scores are summarized in Table 3. Time to follow-up differed significantly between groups (group A: 5.7 (4.7–7.1) years vs. group B: 4.3 (3.0–5.9) years; *P* = 0.001). When comparing the aCS between groups, group B showed a significantly higher score compared to group A (group A: 90 (85.0–91.0) vs. group B: 91 (90.0–93.0); (*P* = 0.037)). There was no significant difference in the QuickDASH score (*P* = 0.384), VAS at rest (*P* = 0.283), and VAS under stress (*P* = 0.286) between the two groups (Table 3).

**Table 3:** Outcome variables.

Outcome Variables	Total	Superior	Anteroinferior	*P* value
Time to follow-up, median (IQR), y	4.9 (3.7–6.1)	5.7 (4.7–7.1)	4.3 (3.0–5.9)	**0.001**^#^
aCS, median (IQR)	91 (86.0–93.0)	90 (85.0–91.0)	91 (90.0–93.0)	**0.037**^#^
QuickDASH, median (IQR)	2.9 (0.6–11.9)	4.6 (0.8–18.5)	2.1 (0–11.3)	0.384^#^
VAS at rest, median (IQR)	0 (0–1)	0 (0–1)	0 (0–2)	0.283^#^
VAS under stress, median (IQR)	1 (0–3)	0 (0–2)	1 (0–3)	0.286^#^
**Implant removal, n (%)**				
Yes	51 (64.6)	17 (60.7)	34 (66.7)	0.630^†^
No	28 (35.4)	11 (39.3)	17 (33.3)	
Time to implant removal, median (IQR), m	14.5 (11–17.6)	17.0 (11.0–23.0)	14.0 (11.0–16.5)	0.154^#^
**Bone union, n (%)**				
Yes	76 (96.2)	27 (96.4)	49 (96.1)	> 0.999^†^
No	3 (3.8)	1 (3.6)	2 (3.9)	
**Return to work, n (%)**				
Yes	75 (94.9)	27 (96.4)	48 (94.1)	> 0.999^†^
No	4 (5.1)	1 (3.6)	3 (5.9)	
**Return to previous workplace, n (%)**				
Yes	63 (91.3)	21 (87.5)	42 (93.3)	0.412^†^
No	6 (8.7)	3 (12.5)	3 (6.7)	

Significant values are written in bold text.

aCS: adjusted Constant score, DASH: Disability of the Arm, Shoulder and Hand, IQR: Interquartile range, VAS: Visual Analog Scale, ^†^: Fisher exact test, ^#^: Mann–Whitney test.

Both groups showed similar implant removal rates without significant differences (group A: 60.7% versus group B: 66.7%; *P* = 0.630) (Table 3). There were no differences in overall implant removal rates between males and females (63.5% vs. 68.8%; *P* = 0.78) and no difference between removal rates for males and females in group A (55.6% vs. 70%; *P* = 0.69) or group B (66.7% vs. 66.7%; *P* =  > 0.99) (Table 4). Additionally, time to implant removal did not differ significantly between groups (*P* = 0.154).

**Table 4:** Implant removal rates by gender and plate position.

Plate position	Male		Female		*P* value
	Removed	Not removed	Removed	Not removed	
Superior, n (%)	10 (55.6)	8 (44.4)	7 (70.0)	3 (30.0)	0.69^†^
Anteroinferior, n (%)	30 (66.7)	15 (33.3)	4 (66.7)	2 (33.3)	> 0.99^†^
Total, n (%)	40 (63.5)	23 (36.5)	11 (68.8)	5 (31.2)	0.78^†^

^†^: Fisher exact test.

At final follow up, 27 (96.4%) patients in group A and 48 (94.1%) patients in group B were able to return to work without significant differences between the two groups (*P* > 0.999). Furthermore, no significant differences were seen with respect to return to the previous workplace (*P* = 0.412) (Table 3).

A total of 76 out of 79 patients (96.2%) had healed at final follow-up. Of those, one patient in group B had a non-union and was revised 412 days after primary surgery, and two patients (one in each group) suffered re-fracture shortly after implant removal in the absence of excessive trauma and were revised 755 and 380 days following the primary surgery, respectively. Demographic data of these patients are demonstrated in Table 5.

**Table 5:** Demographic data of the patients with compromised bony healing.

Group	Sex	Age, y	Comorbidity	Complication	Revision	Time to revision, d	Follow-up, y	Healing	Outcome
A	Male	51	–	Refracture following implant removal	LCP + iliac crest autograft	755	4.8	Yes	DASH 0
									aCS 91
									VAS at rest 0
									VAS under stress 0
B	Female	57	Hashimoto	Refracture following implant removal	LCP + iliac crest autograft	380	3.8	Yes	DASH 5
									aCS 82
									VAS at rest 0
									VAS under stress 0
B	Male	40	–	Non-union	LCP + iliac crest autograft	412	5.7	Yes	DASH 24.2
									aCS 75
									VAS at rest 2
									VAS under stress 6

aCS: adjusted Constant score, DASH: Disability of the Arm, Shoulder and Hand, IQR: Interquartile range, LCP: locking compression plate, VAS: Visual Analog Scale.

Table 6 summarizes complication and revision rates. A total of 13 plate-related complications were found in group A (46.4%) compared to 16 plate-related complications in group B (31.4%); but there was no significant difference between groups (*P* = 0.226). No significant difference was found with regard to revision surgery; a total of seven patients in group A (25%) and five patients in group B (9.8%) underwent revision surgery due to plate related complications (*P* = 0.102). At final follow-up, pain was the most common recorded complication (2 in group A, 7 in group B) followed by postoperative paresthesia (4 in group A and 2 in group B).

**Table 6:** Complications and revisions.

Complications and revisions	Total	Superior	Anteroinferior	*P* value
**Plate-related complications, n (%)**				
Yes	29 (36.7)	13 (46.4)	16 (31.4)	0.226^†^
No	50 (63.3)	15 (53.6)	35 (68.6)	
Pain	9 (11.4)	2 (7.1)	7 (12.1)	
Paresthesia	6 (7.6)	4 (14.3)	2 (3.4)	
Infection	3 (3.8)	1 (3.6)	2 (3.4)	
Implant failure	3 (3.8)	2 (7.1)	1 (1.7)	
Soft-tissue compromise	2 (2.5)	1 (3.6)	1 (1.7)	
Refracture after implant removal	2 (2.5)	1 (3.6)	1 (1.7)	
Hematoma	2 (2.5)	2 (7.1)	0 (0)	
Exostosis	1 (1.3)	0 (0)	1 (1.7)	
Non-union	1 (1.3)	0 (0)	1 (1.7)	
**Revision surgery, n (%)**				
Yes	12 (15.2)	7 (25.0)	5 (9.8)	0.102^†^
No	67 (84.8)	21 (75.0)	46 (90.2)	
Re-osteosynthesis and iliac crest autograft	3 (3.8)	1 (3.6)	2 (3.4)	
Re-osteosynthesis	3 (3.8)	2 (7.1)	1 (1.7)	
Superficial wound revision	2 (2.5)	1 (3.6)	1 (1.7)	
Hemostasis	2 (2.5)	2 (7.1)	0 (0)	
Deep wound revision	1 (1.3)	1 (3.6)	0 (0)	
Debridement of exostosis	1 (1.3)	0 (0)	1 (1.7)	

^†^: Fisher exact test.

## Discussion

The most important finding of this study was that at mid-term follow-up of 4.9 years, implant removal rates for superior plating compared to anteroinferior plating did not differ significantly. This is of relevance since one of the main reasons to choose anteroinferior over superior plating is to optimize soft-tissue coverage and to avoid implant removal.

Prior clinical studies demonstrated higher numbers of implant removal following superior plating, mostly due to symptomatic hardware^8,11^ or cosmetic issues^4,8,9^. In contrast, we found no significant difference in the implant removal rate, and even a trend towards a higher rate in the anteroinferior group. However, our findings are in line with those of Hulsman et al.^22^ who demonstrated a similar removal rate for superior and anteroinferior plates and found that plate positioning was not associated with implant-related irritation. Also, the common assumption, that female patients desire implant removal more frequently was not verified by our study. The overall removal rate, as well as the rates for the superior and anteroinferior group did not show significant differences in female patients when compared to male patients.

In the present study, the anteroinferior plating group reached a significantly higher aCS compared to the superior plating group (*P* = 0.037). However, the minimal clinically important difference (MCID) for the Constant score for patients undergoing rotator cuff repair has been demonstrated to be 10.4 points, thus questioning the clinical significance of this result^25^. This is further emphasized by the fact that no significant differences between anteroinferior and superior plating were found in the other assessed clinical outcome scores. Although the MCID for the Constant score of 10.4 points was established for rotator cuff repair, we are not aware of any studies investigating the MCID for clavicle fractures.

Formaini et al.^8^ evaluated the clinical outcome based on the VAS and the Oxford Shoulder Score. Patients with anteroinferior plating reached a significantly higher score in the OSS (*P* = 0.008), but no differences were found in VAS. This is consistent with our finding of a significantly higher aCS in the anteroinferior group compared to the superior group, but no statistical differences in the VAS. Controversial results were presented by Sohn et al.^21^ who performed a prospective randomized controlled trial to compare minimally invasive plating in the superior versus the anteroinferior position. No differences in CS, pain, strength, range of motion or activities of daily living were observed between the two groups^21^.

In this study, high bone union rates in excess of 95% for both groups were demonstrated. A total of three non-unions where observed, with no significant differences between superior compared to anteroinferior plating. One patient in the anteroinferior group had an obvious non-union that was revised 412 days following primary surgery. The other two patients (one in each group) had their plates removed at an external clinic and sustained a refracture within 1 month following implant removal without adequate trauma. Clinically, this is highly suspicious for non-union as well, although radiographs prior to the hardware removal were not available. For this reason, they were also considered to be a non-union. Overall, these findings are supported by other groups who demonstrated comparable bone union rates for anteroinferior compared to superior plating^8,11,21^.

We observed similar numbers of plate-related complications and revisions in both groups without significant differences. However, a trend towards higher numbers was seen for the superior group. Whereas in the superior group 46.4% of patients had a complication, only 31.4% in the anteroinferior group had a complication. Admittedly, these numbers are high for both groups and the reason may be that our definition for complications was relatively strict (e.g. pain, paresthesia). Consistent with the number of complications, there were also more revisions performed in the superior group (25% vs. 9.8%). Of note, there were two patients in the superior group and one patient in the anteroinferior group that had an implant failure. This may be considered a sign of non-union; however, in these cases implant failure occurred within the first month after surgery and is likely due to patient compliance.

Since our results demonstrated similar outcomes for both groups, we have to reject our hypothesis. In consequence, we recommend choosing the plate position based on patient specific factors and by taking into account surgeon preference. For individuals with limited soft-tissue coverage, the anteroinferior plate position may be advantageous, whereas patients with high physical demands, such as manual laborers or athletes, may benefit from the superior biomechanical stability that has been shown for superior plating^18–20^.

This study is subject to several limitations. First, this study contains bias that is inherent to its retrospective design. The time periods were of different length subsequently leading to the limitation of unequal group sizes. With larger and more similar groups sizes, the observed trends in some outcome parameters may have reached statistical significance. Second, the follow-up period for the superior plate group was longer, thereby subjecting this group to a higher chance of failure. Third, although performed at a single center and thus reducing variability in approach and technique, surgeries were performed by four surgeons. However, all surgeons exclusively performed superior plating in the first period of time, and anteroinferior plating in the second period of time.

## Conclusion

Superior and anteroinferior plating result in high bone union rates and good clinical outcomes with similar rates of plate removal.
